# Adiabatic T2-preparation modules optimized for robustness toward cardiac motion and flow - a comparison with existing techniques at 3 Tesla

**DOI:** 10.1186/1532-429X-13-S1-O14

**Published:** 2011-02-02

**Authors:** Wolfgang G Rehwald, Elizabeth Jenista, Michele A Parker, Han W Kim, Enn-Ling Chen, Raymond J Kim

**Affiliations:** 1 Siemens Healthcare and Duke University, Durham, NC, USA; 2 Duke University Medical Center, Durham, NC, USA

## Objective

To compare two B1-insensitive T2-preparations to existing techniques regarding cardiac motion and flow robustness.

## Background

T2-imaging of the heart at 3 Tesla demands T2-preparations not only insensitive to B0 and B1, but also toward cardiac motion and flow in order to avoid flow artifacts and concomitant myocardial contamination and to preserve endocardial border definition. While more refocusing pulses per T2-prep duration improve robustness, they also increase SAR. We chose four BIREF-1 refocusing pulses (one adiabatic fast passage per pulse) to achieve motion-robustness while requiring lower energy than an equal number of pairs of adiabatic fast-passage. We compared two new techniques (NTs) using described scheme to MLEV4 [1] and adiabatic [2] schemes.

## Methods

**Figure 1:**
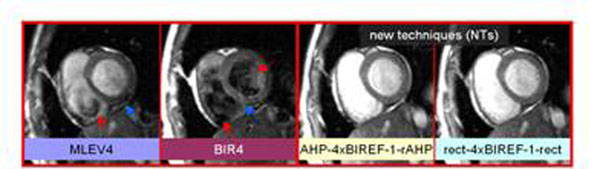
Note the inhomoegeneity of the ventricular cavity for MLEV4 and mBIR4 (red arrows): also note the myocardial artifacts in the same two techniques (blue arrows). Parameters: Flash, flip angle 5°, TW 1.66 mm, TR 4.4 ms, 256x192 matrix. FOV 360x300, mm, 23 segments, thickness 6mm, bandwidth 399Hz/px, trigger pulse 2.

Healthy volunteers (n=18) were scanned (MAGNETOM Verio, Siemens, 32-channel coil, InVivo) using identical parameters (figure 1, T2-prep time 60ms) but different T2-preparations: 1. MLEV4 [1], 2. modified BIR4 (mBIR4, two equal delays between adiabatic half and fast passages [2]), 3. adiabatic half passage (AHP) hyperbolic tangent ±90 pulses and four BIREF-1 [3] refocusing pulses (AHP-BIREF-1x4-rAHP), 4. same refocusing but rectangular instead of AHP pulses (rect-BIREF-1x4-rect). Mid-basal short axis images were acquired and readers (n=2) blinded to the T2-preparation scored the images on a four-point scale for myocardial/cavity homogeneity, endocardial border definition, and flow artifacts. Statistical comparisons were made by ANOVA with Bonferroni correction. We calculated the coefficient of variation (CV) as a measure of inhomogeneity in myocardium and cavity. Signal-to-noise ratio (SNR) and contrast-to-noise ratio (CNR) were measured. The relative module RF energy was calculated using the IDEA environment.

## Results

**Figure 2:**
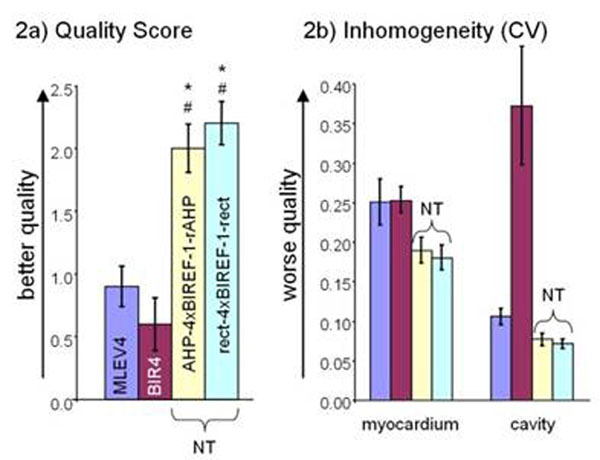
Figure 2a) Both NTs have a statistically significantly better quality score (homogeneity, endocardial border definition, flow artifacts) compared to MLEV4 and mBIR4: *p<0.05 cs MLEV4: #p<0.05 vs mBIR4 (score: 0 poor, 1 equivocal, 2 satisfactory, 3 excellent). Figure 2b) Note the decreased inhomogeneity (i.e. improved homogeneity) of both NTs in myocardium and vavity compared to MLEV4. Also see the drastically increased inhomogeneity of mBIR4 in the cavity.

Both NTs resulted in higher image quality and reduced inhomogeneity (figure 1). Significantly better quality scores were obtained with AHP-BIREF-1x4-rAHP (2.0±0.19, mean±sem) and rect-BIREF-1x4-rect (2.2±0.17) compared to MLEV4 (0.9±0.61) and mBIR4 (0.6±0.21) (figure 2a). Less inhomogeneity was observed with both NTs in myocardium and cavity relative to MLEV and mBIR4 (figure 2b). mBIR4 had particularly poor homogeneity in the ventricular cavity often resulting in partially dark blood appearance. Myocardial SNR was better in the NTs (20.8±1.5 MLEV4, 19.9±0.9 mBIR4, 26.6±1.4 AHP-BIREF-1x4-rAHP, 25.8±1.6 rect-BIREF-1x4-rect). CNR was also higher in the NTs (31.6±2.1, 11.6±3.5, 36.4±1.9, 35.2±2.0, order as above). For all modules, the energy relative to MLEV4 was higher, specifically 241% (mBIR4), 541% (AHP-BREF1x4-rAHP), and 490% (rect-BIREF-1x4-rect), but did not exceed SAR limits.

## Conclusions

Two new T2-preparation techniques for imaging the heart in vivo have been developed with significantly better performance than existing techniques.
